# Discretizing continuous variables in nutrition and obesity research: a practice that needs to be cut short

**DOI:** 10.1038/s41387-023-00248-0

**Published:** 2023-11-08

**Authors:** Osvaldo F. Morera, Mosi I. Dane’el, Brandt A. Smith, Alisha H. Redelfs, Sarah L. Ruiz, Kristopher J. Preacher, Leah D. Whigham

**Affiliations:** 1 Department of Psychology, University of Texas at El Paso, 500 W. University Ave., El Paso, TX 79968 USA; 2 State of New Mexico, New Mexico Children, Youth and Families Department, 4775 Indian School Rd, Albuquerque, NM 87110 USA; 3 Department of Psychology, Columbus State University, Arnold Hall 272, 4225 University Avenue, Columbus, GA 31907 USA; 4 Department of Public Health, Brigham Young University, 2136 LSB, Provo, UT 84602 USA; 5 Interdisciplinary Health Sciences Program, University of Texas at El Paso, 500 W. University Ave., El Paso, TX 79968 USA; 6 Department of Psychology and Human Development, Vanderbilt University, Peabody #552, 230 Appleton Place, Nashville, TN 37203 USA; 7 Center for Community Health Impact, Department of Health Promotion and Behavioral Sciences, The University of Texas Health Science Center at Houston School of Public Health, 5130 Gateway East Blvd., MCA 114, El Paso, TX 79905 USA

**Keywords:** Epidemiology, Nutrition

## Abstract

**Background/objectives:**

Nutrition and obesity researchers often dichotomize or discretize continuous independent variables to conduct an analysis of variance to examine group differences. We describe consequences associated with dichotomizing and discretizing continuous variables using two cross-sectional studies related to nutrition.

**Subjects/methods:**

Study 1 investigated the effects of health literacy and nutrition knowledge on nutrition label accuracy (*n* = 612). Study 2 investigated the effects of cognitive restraint and BMI on fruit and vegetable (F/V) intake (*n* = 586). We compare analytic approaches where continuous independent variables were either discretized/dichotomized or analyzed as continuous variables.

**Results:**

In Study 1, dichotomization of health literacy and nutrition knowledge for 2 × 2 ANOVA revealed health literacy had an effect on nutrition label accuracy. Nutrition knowledge has an effect on nutrition label accuracy, but the health literacy by nutrition knowledge interaction was not significant. When analyzed using regression, the nutrition knowledge effect was significant. The simple effect of health literacy was also significant when health literacy equals zero. Finally, the quadratic effect of health literacy was negative and significant. In Study 2, dichotomization and discretization of cognitive restraint and BMI were used for three ANOVAs, which discretized BMI in three ways. For all ANOVAs, the BMI main effect for predicting fruit and vegetable intake was significant, the interaction between BMI and cognitive restraint was non-significant, and cognitive restraint was only significant when both variables were dichotomized. When analyzed using regression, the continuous mean-centered variables, and their interaction each significantly predicted F/V intake.

**Conclusions:**

Dichotomizing continuous independent variables resulted in distortions of effect sizes across studies, an inability to assess the quadratic effect of health literacy, and an inability to detect the moderating effect of BMI. We discourage researchers from dichotomizing and discretizing continuous independent variables and instead use multiple regression to examine relationships between continuous independent and dependent variables.

## Introduction

Studies in nutrition and obesity science routinely involve continuous variables, such as waist circumference, body mass index (BMI), and various eating indices. Often, these variables are *dichotomized* to create groups of individuals who are “high” or “low” on the continuous measure. For example, researchers will employ a median split to create two groups based on a continuous variable. A recent Google Scholar search for publications since 2020 for the phrase “median split” and “obesity” found 1350 results. Further, the term “median split” occurred at least 15 times in the *Proceedings of the National Academy of Sciences* between the years 2020 and 2023.

While dichotomization often occurs at the median, it can occur at any point in a continuous distribution. Moreover, continuous variables are often *discretized* into more than two categories. The Centers for Disease Control (CDC) [1] classifies BMI as underweight <18.5, healthy weight between 18.5 and 24.9, and overweight 25.0– 29.9. Obesity is BMI > 30, with further breakdown into Class 1 (30 to <35), Class 2 (35 to <40), and Class 3 (≥40). The following discussion is important for researchers who dichotomize or discretize continuous variables.

We briefly review the problems with dichotomizing and discretizing continuous variables and propose more effective methods using common, and often free, software. Two examples follow that demonstrate the negative consequences of dichotomization and discretization. We recommend solutions that do not require more than a basic understanding of multiple linear regression and moderated regression procedures. Most of the alternatives have been integrated into common statistical packages (e.g., SAS, SPSS, R, and Python). Further, online tools to probe interactions for moderated regression are readily available.

### Issues associated with dichotomizing and discretizing continuous independent variables

This issue has been a concern in other scientific fields. Leading journals [2–7] have recommended against the use of dichotomizing continuous variables. An exchange in the *Journal of Consumer Psychology* [8–12] on the use of the median split highlights many of these issues. However, we will discuss the most significant concerns as they pertain to the obesity and nutrition fields.

The negative consequences of dichotomizing and discretizing continuous independent variables are well known. Cohen [13] demonstrated that effect sizes may be attenuated when continuous normally distributed independent variables are dichotomized at the mean. The reduction of the correlation also worsens if dichotomization takes place further from the mean [13]. One reason why effect sizes will be attenuated is due to the loss of information which results when scores that differ on the continuous variable are treated the same because they fall above (or below) an arbitrary value (e.g., when they fall within a specific range or category). This loss of information adds measurement error to the assessment of the predictor variable. MacCallum and colleagues [14] also showed that dichotomizing continuous independent variables will reduce the reliability of the measures used in laboratory and field experiments. Reduced reliability results in the attenuation of the relationship between two variables, i.e., a decreased likelihood that a difference between groups will be detected.

MacCallum et al. [14] also conducted simulation studies (see Table 1 in their paper) to determine the proportion of times when dichotomizing normally distributed random variables may also result in spurious *increases* in the correlation between the dichotomized variable and the outcome variable due to sampling error. These authors found that spurious increases were more likely to occur when the correlation between the two variables was low (correlations equal to 0.10) and when sample size was lower (sample sizes of 50). Even in samples as large as 300 and with a correlation of 0.10, the MacCallum, et al. [14] simulation studies demonstrated that these spurious increases in the correlation due to dichotomization occurred over 28% of the time.

There are still other reasons why researchers should not dichotomize or discretize continuous independent variables. As statistical power is defined as the probability of detecting an effect of a certain size if the effect is there, procedures that distort effect sizes will also distort the power of those statistical tests. Moreover, distorted effect sizes further impact future meta-analyses, thus impacting the generalizability of the effect in question.

In addition, Maxwell and Delaney [15] demonstrated that one may also obtain spurious statistical results when multiple variables are dichotomized in the same analyses. In other words, one may find a main effect in ANOVA that is statistically significant as a result of dichotomization at the median, but the regression analysis may not reveal this effect. These spurious main effects or interactions are more likely to arise as the correlations among the independent variables increase. In addition, the practice of dichotomizing and discretizing continuous variables may lead to biased parameter estimates. These results may not be replicated and may lead researchers to make incorrect conclusions. Furthermore, one cannot examine nonlinear relationships between continuous independent variables and outcome variables if continuous variables are dichotomized.

## Analytic approach to demonstrations

We compare analytic approaches from two studies where continuous independent variables were either discretized/dichotomized or analyzed as continuous variables. Since the correlations among independent variables may inflate Type I error rates and lead to the detection of spurious results [15], we report the correlations among the predictors. We also report the correlations between the predictors and criterion, as research shows dichotomization may both attenuate and inflate effect sizes [13, 14]. In addition, we test models for nonlinearity, as prior research has shown that un-modeled nonlinear relationships may create a spurious interaction in linear regression analysis [16, 17]. Both studies were approved by the University of Texas at El Paso Institutional Review Board, and participants consented prior to participation.

In Study 1, an un-modeled quadratic effect would have resulted in a two-way interaction with multiple linear regression. We present the model with the quadratic effect below and compare it to analyses associated with dichotomizing continuous independent variables. Since the predictors could take on the value of zero and zero represented a meaningful score of not answering any item correct on the measures, the predictors were not centered. In Study 2, there was no quadratic effect for either predictor. Therefore, we compare a linear regression model with a two-way interaction to a variety of ANOVA models. In the regression analyses in Study 2, we mean-centered the predictors as neither predictor could take on the value of zero. Data from Study 1 is available from the first author, while data from Study 2 is available from the last author.

### Study 1: Materials and methods

Our first example uses three variables to examine the relationship between nutrition knowledge and health literacy. The relationships among accurately reading food labels, nutrition knowledge, and health literacy (*n* = 612) as part of a larger model were explored. Prior to the start of the study, informed consent was obtained from all participants. Participants were female (71.4%) with an average age of 20.26 years (SD = 3.89) and Latinx (85.3% of the sample). Data were collected online in Qualtrics during 2017–2018 academic school year. The study had sufficient power: Assuming that health literacy, nutrition knowledge and a quadratic effect of health literacy explained 12.5% of variability in food label accuracy scores and the quadratic effect of health literacy uniquely explained 2.5% of unique variability, a sample of 385 participants would be needed to detect the quadratic effect with a Type I error rate of 0.05 and power = 0.80 [18] This study was not replicated.

To measure health literacy, we used a modified version of the Health Literacy Skills Instrument [19, 20] with items being scored as correct or incorrect. The composite score represents the total correct answers. Scores range from 0 to 9, with higher scores indicating greater health literacy. Participants’ average score was 5.61 (SD = 1.54) with reliability (indexed by KR-20) of 0.68, 95% CI (0.64, 0.72). In order to create two categories, anyone scoring 6 or lower was considered “low” on health literacy (68.8% of the sample) and anyone scoring 7, 8, or 9 was considered “high.” While it was not possible to create approximately equal-sized groups with discrete outcomes that assume the values between 0 and 9, the arbitrary choice of where to dichotomize can be seen as an additional impediment to valid inference when dichotomizing independent variables.

For our second variable, we measured nutrition knowledge using a modified version of a measure developed by Parmenter and Wardle [21]. The measure consisted of 20 items that pertain to the relationship between diet and health problems. The composite score represents the total number of correct answers. Participant scores ranged from 0 to 18 with an average score of 10.73 (SD = 3.26) and a reliability estimate (indexed by KR-20) of 0.65, 95% CI (0.61, 0.69). To create two artificial categories, scores were dichotomized at 12, where participants who scored 11 or lower were “low” on nutrition knowledge (56.4% of the sample).

To measure nutrition label accuracy, a modified version of the Nutrition Label Survey [22] based on our earlier work [19] was used. Participant scores ranged from 0 to 16 with an average score of 10.86 (SD = 3.29). The reliability of the scores for this measure (as indexed by KR-20) equaled 0.77, 95% CI (0.74, 0.79).

### Study 1: Results

#### Univariate relationship between predictors and label accuracy

The relationship between health literacy and label accuracy as a continuous variable is *r*(610) = 0.30, *P* < 0.001. Health literacy explains 8.8% of the variability in label accuracy scores. The relationship between dichotomized health literacy and label accuracy is attenuated, as the correlation was *r*(610) = 0.14, *P* < 0.001, explaining 2.0% of the variance. This example demonstrates a dramatic 77.2% reduction in effect size when using dichotomized variables (8.8% vs. 2.0%).

In addition, the correlation between nutrition knowledge and label accuracy was *r*(610) = 0.29, *P* < 0.001 and explains 8.3% of the variance in label accuracy scores. The correlation between the dichotomized nutrition knowledge and label accuracy was *r*(610) = 0.20, *P* < 0.001, and the proportion of variance explained was 4.1%. Finally, the two predictors were moderately correlated, *r*(610) = 0.30, *P* < 0.001.

#### Results of the analysis of variance with dichotomized independent variables

In this demonstration, we investigated the effects of dichotomized continuous variables on label accuracy scores. The main effect for nutrition knowledge was statistically significant, *F*(1, 608) = 14.27, *P* < 0.001, squared partial correlation = 0.023, Cohen’s *d* = 0.43. The nutrition label accuracy score (mean ± SE) for the participants with “low” vs. “high” nutrition knowledge was 10.59 ± 0.20 vs. 11.66 ± 0.20.

The main effect for health literacy was statistically significant, *F*(1, 608) = 8.44, *P* = 0.004, squared partial correlation = 0.014, Cohen’s *d* = 0.31. The nutrition label accuracy score (mean ± SE) for the participants with “low” vs. “high” health literacy was 10.72 ± 0.16 vs. 11.53 ± 0.23. Finally, the interaction was not statistically significant, *F*(1, 608) = 3.512, *P* = 0.061, squared partial correlation = 0.006. The *R*^2^ for this analysis equaled 0.06, indicating that the dichotomized independent variables and their interaction explain 6% of the label accuracy variability.

#### Results of the multiple regression analysis

We also analyzed the data using multiple regression. Initially, we regressed label accuracy on nutrition knowledge, health literacy, and their interaction. As prior research [16, 17] shows that un-modeled nonlinear effects may result in spurious interactions, we also regressed label accuracy on nutrition knowledge, health literacy, and health literacy squared (to estimate a quadratic effect). This model was a better model in terms of the proportion of variance explained. Another model containing the quadratic effect for health literacy and a two-way interaction between health literacy and nutrition knowledge was also examined, but the two-way interaction was not significant. We now discuss the regression model with the quadratic effect.

In the analysis with the quadratic effect, the *R*^2^ equaled 0.17, almost three times the proportion of variance explained in the prior ANOVA. Moreover, the partial regression coefficient for nutrition knowledge was significant: for every 1-unit increase in nutrition knowledge, the predicted label accuracy score increased 0.17 points (*P* < 0.001) holding health literacy constant. The squared partial correlation coefficient associated with this variable equaled 0.030, which indicates that nutrition knowledge uniquely accounts for 3.0% of the unexplained variability in nutrition label accuracy scores that is not accounted for by the other predictors in the model.

The simple effect for health literacy when health literacy equals zero is 2.52 (*P* < 0.001) showing that when health literacy is low, small differences in health literacy are associated with large differences in predicted label accuracy scores. The squared partial correlation coefficient associated with this simple effect equaled 0.07, indicating that this variable uniquely accounts for 7.0% of the unexplained variability in nutrition label accuracy scores that is not accounted for by the other predictors in the model.

One obvious shortcoming of dichotomizing health literacy for an ANOVA is the inability to examine nonlinear relationships. Put another way, the power to detect this quadratic effect in an ANOVA equals zero. In this model, the quadratic effect was statistically significant. At the point where Health Literacy = 0 (its minimum), and holding constant Nutrition Knowledge, a 1-unit increase in Health Literacy translates to a 2.52-point increase in predicted Label Accuracy when health literacy equals zero, and −0.20 is half the amount by which this effect changes for every 1-unit increase in Health Literacy thereafter. So, the initially significant positive effect of Health Literacy weakens as Health Literacy increases (*P* < 0.001). The squared partial correlation coefficient associated with this quadratic effect equaled 0.049, indicating this variable uniquely accounts for 4.9% of the unexplained variability in nutrition label accuracy scores that is not accounted for by other predictors are in the model.

#### Probing the quadratic effect

For our model,

$$\hat{y}=1.53+0.17\,{Nutrition}\,{Knowledge}+2.52\,{Health}\,{Literacy}-0.20\,{Health}\,{Literac}{y}^{2}$$

Re-expressing this model,

$$\hat{y}=1.53+0.17\,{Nutrition}\,{Knowledge}+2.52\,{Health}\,{Literacy}-0.20\,({Health}\,{Literacy}* {Health}\,{Literacy})$$

The quadratic effect above indicates the quadratic effect is an interaction, i.e., the regression of accuracy in reading nutrition labels on health literacy depends on where you stand on health literacy. Rearranging terms, this model can be re-expressed as:

$$\hat{y}=1.53+.17\,{Nutrition}\,{Knowledge}+\,\left(2.52-0.20\,{Health}\,{Literacy}\right){Health}\,{Literacy}$$

To aid in the interpretation of the parenthesized term, we used the Johnson–Neyman [23] regions of significance approach to determine what values of health literacy made the parenthesized term statistically significant. As Spiller and colleagues [24] highlight, the choice of what values equal zero among the predictor variables must be kept in mind when examining the parenthesized term above.

There are several ways to probe an interaction, including the use of 3D graphs [25], spotlighting or pick-a-point [16], floodlighting [26], and the Johnson–Neyman [23] regions of significance approach. The use of 3D graphs allows for a three-dimensional examination of the relationship between the predictor variables and the dependent variable. Spotlighting or pick-a-point assesses the statistical significance of the parenthesized term at a particular value of the moderator variable. Floodlighting examines all possible values that the moderator variable can take on in the parenthesized term for the simple slope, while the Johnson–Neyman regions of significance approach provides a range of the values for the moderator where the parenthesized term for the simple slope is statistically significant. We chose to use the Johnson–Neyman approach, as the resulting graph is easy to interpret and provides the regions of significance.

**Fig. 1:**
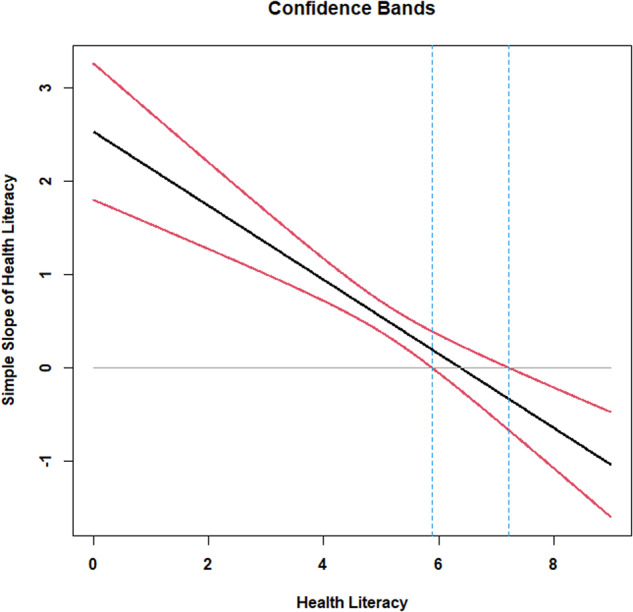
Johnson-Neyman Regions of Significance Plot for Study1. Probing the quadratic effect of health literacy.

Miller et al. [27] provide online tools that involve the use of the Johnson–Neyman approach to examine the statistical significance of the parenthesized term when the linear model contains a quadratic effect. Using these tools, the parenthesized term above is not statistically significant when health literacy ranges between 5.8883 to 7.2203. In other words, when health literacy is equal to 6 or 7, the local linear effect of health literacy on accuracy in reading nutrition labels is not statistically significant. Between the values of 0 and 5, the local linear effect of health literacy on accuracy in reading food labels is positive. For the individuals who score either 8 or 9, the local linear effect of health literacy on accuracy in reading food label scores is negative. Figure 1 depicts this simple slope.

### Study 2: Materials/subjects and methods

Our second example examines the relationships among cognitive restraint, BMI, and fruit & vegetable (F/V) intake (*n* = 586). Participants were female (66.2%), Latinx (69.3%), and had annual income less than 50 K (53.6%). The average age was 35.5 (SD = 14). Data were collected from health professionals, nutrition students, and community members from March 2018 to June 2019. Prior to the start of the study, informed consent was obtained from all participants. The study had sufficient power: Assuming that the interaction between cognitive restraint and BMI explained 1.5% of unique variability and that BMI, cognitive restraint, and their interaction explained 12.5% of the variability in F/V intake, a sample of 462 participants would be needed to detect this interaction with a Type I error rate of 0.05 and power = 0.80 [18]. This study was not replicated.

To measure cognitive restraint, we used a modified version of this domain from the Three Factor Eating Questionnaire [28, 29] with items being scored on a 1–4 Likert scale. Mean composite scores range from 1 to 4, with increased scores indicating greater cognitive restraint. Participants’ average score was 2.61 (SD = 0.56) with reliability (indexed by coefficient alpha) of 0.67. For the purpose of this example, anyone scoring 2.60 or lower was considered “low” on cognitive restraint and anyone scoring above 2.60 as considered “high” on cognitive restraint. “Low” scoring individuals made up 45.9% of the sample.

For our second variable, we calculated the participants’ BMI. Participant height (Seca 213 stadiometer, Hamburg, Germany) and weight were measured (InBody 270 and InBody 570 Body Composition Analyzers, Seoul, South Korea), and BMI was calculated. Heights were rounded to the nearest half-centimeter. Participant BMIs ranged from 17.0 to 60.7 with an average score of 27.99 (SD = 6.05). Several artificial groupings were created: dichotomized at median, discretized per CDC guidelines [1], and dichotomized as having obesity or not. To create two artificial categories, scores were dichotomized at 27.1, where participants who scored 27.1 or lower were “low” BMI and were assigned a score of 0. “Low” scoring participants made up 50.2% of the sample. Individuals whose BMI was greater than 27.1 were considered “high” and were assigned a score of 1.

In a separate analysis, BMI was discretized at the following points, according to CDC guidelines [1]: underweight, BMI < 18.5 (1.2% of the sample); healthy weight, BMI 18.5–24.9 (32.4% of the sample); overweight, BMI 25.0–29.9 (36% of the sample); Class 1 Obesity, BMI 30–34.9 (18.4% of the sample); Class 2 Obesity, BMI 35–39.9 (7% of the sample); and Class 3 Obesity, BMI ≥ 40 (4.9% of the sample). In a third ANOVA, BMI was discretized using modified CDC guidelines where Class 1, Class 2, and Class 3 obesity were merged into an “obesity” category, constituting 30.3% of the sample.

For F/V intake, we measured skin carotenoid levels, a biomarker for total F/V intake, using reflectance spectroscopy (VEGGIE METER® by Longevity Link Incorporated, Salt Lake City, UT, USA) [30, 31]. Participant scores ranged from 29 to 709 with an average score of 275.67 (SD = 110.149).

### Study 2: Results

#### Univariate relationship between predictors and F/V intake

When analyzed as a continuous variable, the relationship between BMI and F/V intake is *r*(584) = −0.18, *P* < 0.001. BMI explains 3.1% of the variability in F/V intake scores. The relationship between a dichotomized BMI and F/V intake increased, with a correlation of *r*(584) = −0.21, *P* < 0.001, explaining 4.2% of the variance. As mentioned earlier, one of the conditions under which dichotomizing continuous variables may increase the correlation with the criterion occurs when the correlation is small, as is the case in this example. These correlations do not statistically differ from one another, *Z* = 1.07, *P* = 0.29. [32, 33].

The correlation of cognitive restraint as a continuous variable with F/V intake was *r*(584) = 0.13, *P* = 0.001 while the correlation of dichotomized cognitive restraint with F/V intake was *r*(584) = 0.14, *P* = 0.001. The correlations with these two approaches did not statistically differ from one another, *Z* = −0.17, *P* = 0.86 [32, 33]. Finally, the correlation between continuously measured cognitive restraint and continuously measured BMI was *r*(584) = 0.04, *P* = 0.28.

#### Results of the analysis of variance with discretized and dichotomized independent variables

To investigate the effects of dichotomized and discretized continuous variables on F/V intake scores we conducted the following ANOVAs on F/V intake: 2 (low vs high cognitive restraint) × 2 (low vs high BMI), a 2 (low vs high cognitive restraint) × 4 (underweight–healthy weight–overweight-obesity), and a 2 (low vs high cognitive restraint) × 6 (underweight–healthy weight–overweight-Class 1 Obesity-Class 2 Obesity-Class 3 Obesity).

For the 2 × 2 ANOVA on F/V intake scores, the main effect of BMI was statistically significant, *F*(1, 582) = 24.58, *P* < 0.001, squared partial correlation = 0.041. The F/V score (mean ± SE) for the participants with low vs. high BMI was 296.26 ± 6.24 vs. 252.42 ± 6.26. The main effect for cognitive restraint was statistically significant, *F*(1, 582) = 12.07, *P* < 0.001, squared partial correlation = 0.02. The scores (mean ± SE) for participants with low vs. high cognitive restraint was 258.98 ± 6.50 vs. 289.70 ± 5.99. Finally, the interaction was not statistically significant, *F*(1, 582) = 3.34, *P* = 0.062, squared partial correlation = 0.006. The *R*^2^ for this analysis equaled 0.067, indicating that the dichotomized independent variables and their interaction explain 6.7% of the variability in F/V intake. For these analyses, one would conclude that individuals with low BMI (relative to high BMI), and that those individuals with high cognitive restraint (relative to low cognitive restraint), consume more F/Vs.

For the 2 × 4 ANOVA (respectively, low vs. high cognitive restraint; underweight–healthy weight–overweight–obesity) on F/V intake scores, the main effect for BMI was statistically significant, *F*(3, 578) = 6.94, *P* < 0.001, squared partial correlation = 0.012. The F/V score (mean ± SE) for participants in the categories of underweight, healthy weight, overweight, and obesity were 246.50 ± 44.83, 302.78 ± 7.77, 267.4 ± 7.52, 254.27 ± 8.05, respectively. Bonferroni contrasts revealed that individuals with healthy weight ate more F/Vs than participants with overweight and obesity. The main effect for cognitive restraint was not statistically significant, *F*(1, 578) = 3.23, *P* = 0.073, squared partial correlation = 0.006. Finally, the interaction was also not statistically significant, *F*(3, 578) = 2.07, *P* = 0.103, squared partial correlation = 0.004. The *R*^2^ for this analysis equaled 0.065, indicating that the discretized predictors and their interaction explain 6.5% of F/V intake variability.

For the 2 (low vs. high cognitive restraint) × 6 (underweight–healthy weight–overweight–obesity 1–obesity 2–obesity 3) ANOVA on F/V intake scores, the main effect for BMI was statistically significant, *F*(5, 574) = 4.85, *P* < 0.001, squared partial correlation = 0.008. The F/V score (mean ± SE) for participants in the categories of underweight, healthy weight, overweight, obesity 1, obesity 2, and obesity 3 were 246.50 ± 44.81, 302.78 ± 7.77, 267.43 ± 7.52, 263.94 ± 10.42, 239.50 ± 16.77, and 232.98 ± 19.90, respectively. Bonferroni contrasts revealed that healthy-weight individuals ate more F/Vs than those with overweight and all participants with obesity. The main effect for cognitive restraint was not statistically significant, *F*(1, 574) = 1.42, *P* = 0.23, squared partial correlation = 0.002. Finally, the interaction was also not statistically significant, *F*(5, 574) = 1.72, *P* = 0.128, squared partial correlation = 0.003. The *R*^2^ for this analysis equaled 0.072, indicating that the discretized predictors and their interaction explain 7.2% of the F/V intake variability.

#### Results of the multiple regression analysis

We also analyzed the data using multiple regression, where BMI and cognitive restraint and their interaction were included in the model. In general, the regression model in our example can be expressed as:

$$\hat{y}={\beta }_{0}+{\beta }_{1}X+{\beta }_{2}Z+{\beta }_{3}{XZ}$$

Rearranging terms and declaring X (cognitive restraint) as the focal predictor and Z (e.g., BMI) as the moderator variable yields:

$$\hat{y}={(\beta }_{0}+{\beta }_{2}Z)+{({\beta }_{1}+\beta }_{3}Z)X$$

The first parenthesized term is known as the *simple intercept*; we see that the simple intercept is dependent on the value of Z, the moderator, and its associated conditional partial regression coefficient, ${\beta }_{2}$. The second parenthesized term is known as the simple slope, and it is also dependent on the value of Z. Researchers often want to provide “meaning” to ${\beta }_{1}$, so that one can say that for every 1 unit change in X, ${\beta }_{1}$ will represent how much the predicted outcome variable will change. That claim can be made only when Z = 0. In the current example, BMI and cognitive restraint can never take on the value of zero. As a result, ${\beta }_{1}$ represents an effect that has no meaning. The above equation can also be rearranged making Z the focal predictor, so that the meaningfulness of ${\beta }_{2}$ will depend on whether X (e.g., cognitive restraint) can assume the value of 0.

McClelland et al. [34] provide an easy-to-understand synopsis of a variety of ways to provide meaning to these conditional partial regression coefficients. Some of these methods involve mean-centering the predictor variables [35] and performing orthogonal transformations of the predictor variables [36, 37]. As McClelland et al. [34] point out, these transformations of the predictor will not alter the estimate of ${\beta }_{3}$ or its associated standard error. In addition, the semi-partial correlation and partial correlation involving the interaction term and the outcome will not be changed due to either mean centering or the use of an orthogonal transformation. These transformations will also have no effect on model fit [38]. The primary benefit of transforming predictor variables is to provide “meaning” to ${\beta }_{1}$ and ${\beta }_{2}$.

It is also clear that interpreting the simple slope depends on the numeric values of ${\beta }_{1}$ and/or ${\beta }_{2}$ [24], which depends on how predictor variables are transformed. In this demonstration, we decided to mean center BMI and cognitive restraint. In this analysis, the *R*^2^ equaled 0.059, which is less than estimates of *R*^2^ from the ANOVA models. The conditional effect for a centered BMI was statistically significant, Β = −3.75 (SE = 0.76), *t* = −4.95, *P* < 0.001. The squared partial correlation coefficient associated with this conditional effect equaled 0.040, indicating that this variable uniquely accounts for 4% of the unexplained variability in F/V intake scores that is not accounted for by the other predictors.

The conditional effect for mean cognitive restraint was significant: Β = 24.22 (SE = 8.01), *t* = 3.02, *P* = 0.003. The squared partial correlation coefficient associated with this variable equaled 0.015, which indicates that cognitive restraint uniquely accounts for 1.5% of the unexplained variability in F/V intake that is not accounted for by the other predictors. The conditional effect of cognitive restraint is qualified by an interaction with BMI: Β = −2.84 (SE = 1.34), *t* = −2.12, *P* = 0.034. These findings contradict what was found with the various ANOVA models where cognitive restraint was dichotomized and BMI was either dichotomized or discretized. The squared partial correlation coefficient associated with this conditional effect equaled 0.008, indicating this variable uniquely accounts for 0.8% of the unexplained variability in F/V intake scores that is not accounted for by the other predictors.

#### Probing the interaction

As discussed earlier, once an interaction is found in regression, the interaction needs to be understood. In the equations below, $\hat{y}$ will denote the predicted F/V intake score. For our model,

$$\hat{y}=276.10+24.22({Mean}-{centered}\,{Cognitive}\,{Restraint})+-3.75({Mean}-{centered}\,{BMI})-2.84(\left({Mean}-{centered}\,{Cognitive}\,{Restraint}\right)* \left({Mean}-{centered}\,{BMI}\right))$$

This model is a moderated multiple regression equation, where one independent variable is the focal predictor and the other independent variable is the moderator. For this example, cognitive restraint will be the focal predictor of F/V intake and BMI moderates the relationship between cognitive restraint and F/V intake. Rearranging terms, this model can be re-expressed as:

$$\hat{y}=\left(276.10+-3.75({mean}-{centered}\,{BMI})\right)+\left(24.22+-2.84\left({mean}-{centered}\,{BMI}\right)\right)({mean}-{centered}\,{Cognitive}\,{Restraint})$$

To aid in the interpretation of this model, many researchers would dichotomize the moderator and plot the regression of the outcome variable on the focal predictor separately for individuals who are “low” and “high” on the moderator. Other researchers might pick three arbitrary points of the moderator variable (e.g., the mean and 1 standard deviation above and below the mean of the moderator variable) and plot the regression of the dependent variable on the focal predictor at these three points. While such procedures are commonly used, they do not determine the numeric values of the moderator variable that make the simple slope statistically significant. Moreover, such an approach limits generalizability as the values of the mean and the standard deviation are sample-dependent [24].

**Fig. 2:**
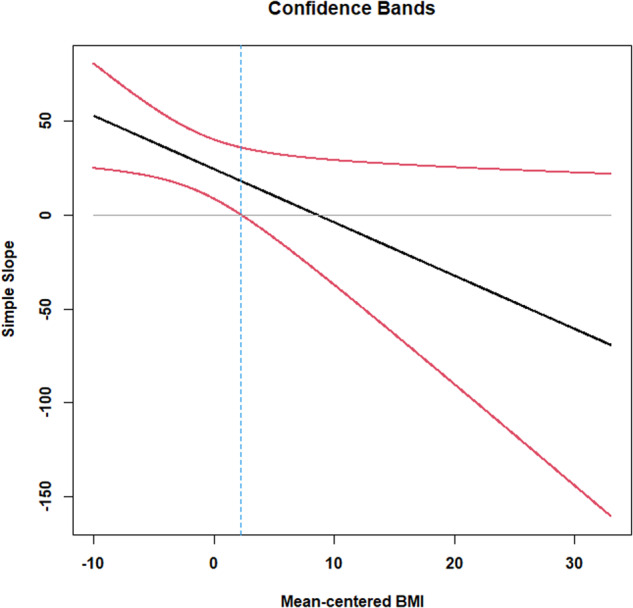
Johnson-Neyman Regions of Significance Plot for Study2. Probing the two-way interaction with mean-centered BMI as the moderator variable.

The Johnson–Neyman technique [23] allows for such an assessment by creating 95% confidence intervals for a simple slope for all hypothetical values of the moderator. Confidence limits that exclude zero indicate the simple slope is statistically significant at that value of the moderator. In Fig. 2, the vertical axis consists of values of the simple slope, while the horizontal axis are the numeric values of the moderator variable, BMI. Looking at Fig. 2, we see the values of BMI slightly above 2.24 units above the mean have confidence intervals that contain 0. Using PROCESS [39], the simple slope is not statistically significant at mean-centered BMI values above 2.24 units above the mean. Mean BMI in this sample equaled 27.99. In practical terms, the conditional effect of increased mean-centered cognitive restraint on F/V intake is detectable for participants classified as underweight, healthy weight, overweight, and for some who would be classified as having obesity (BMI < 30.23, which is 27.99 + 2.24). For participants who have BMIs >30.23, there is no significant association between F/V intake and cognitive restraint.

As interactions are symmetric, we can also treat cognitive restraint as the moderator variable. Rearranging the above expression,

$$\hat{y}=\left(276.10+24.22({mean}-{centered}\,{Cognitive}\,{Restraint}\right)+\left(-3.75+-2.84\left({mean}-{centered}\,{Cognitive}\,{Restraint}\right)\right)({mean}-{centered}\,{BMI})$$

Using the Johnson–Neyman [23] regions of significance approach, the simple slope above is statistically significant when cognitive restraint scores are greater than or equal to 0.64 units below the mean on cognitive restraint.

## Discussion

Researchers often create dichotomized/discretized groups so that they can conduct an ANOVA or an independent samples *t*-test to assess group differences or help them interpret an interaction. Researchers will also dichotomize a continuous independent variable and use those groupings to interpret an interaction in a moderated multiple regression. As both of those options also come with problems mentioned earlier, we recommend that researchers keep independent variables continuous and use multiple linear regression.

One may argue that there are situations to dichotomize or discretize continuous independent variables. For example, if there is reason to suspect that there are underlying groupings for continuous independent variables, researchers should use alternative strategies like latent class analysis or taxometric techniques. These techniques can be used to examine underlying groupings rather than impose an arbitrary, sample-specific cut point like the median. [14] Spiller and colleagues [24] also mention that simple slopes that result from a multiple linear regression of continuous variables may be probed at established cut points. For example, the United States Armed Forces [40] uses established cut points to make enlistment decisions and decisions to provide recruiting bonuses to individuals who score very well on the Armed Services Vocational Aptitude Battery. It is worth emphasizing that these cut scores are established and not sample-specific.

Online tools and software macros exist to assist in interpreting interactions without the need to dichotomize one or more of the variables [39, 41]. For example, http://www.quantpsy.org has tools that allow for the plotting of two- and three-way interactions in multiple regression. Andrew Hayes developed the PROCESS [39] macro for SAS and SPSS, and the “processr” package for R which allow for an examination of interactions. These tools use the Johnson–Neyman [23] technique to solve for numeric values of the moderating variable for which the simple slope is statistically significant. These techniques allow researchers to better interpret their interactions compared to dichotomizing continuous independent variables.

We demonstrated across two different studies a number of adverse effects of dichotomizing and discretizing continuous independent variables. In the first example, we were unable to model a nonlinear relationship. In addition, correlations were attenuated, and model fit was reduced by almost two-thirds when continuous predictor variables were dichotomized. In a second example involving the uncorrelated predictors of cognitive restraint and BMI, we witnessed the negative effects of dichotomizing these variables in the inability to detect the interaction in the 2 × 2 ANOVA. In fact, none of the reported ANOVAs detected the interaction.

With the examples above, we demonstrated that multiple regression is the preferred analytic procedure. No reason for dichotomizing or discretizing independent variables can compete with the adverse effects discussed in this report. In our examples, we saw that dichotomizing and discretizing continuous independent variables resulted in ANOVA models that did not detect a quadratic effect or an interaction between the predictor variables. The quadratic effect and the interaction was probed using the Johnson–Neyman [27] regions of significance to accurately describe the nature of the relationship.

Correctly analyzing data is not only an empirical issue but also an ethical issue. Methodologists [42, 43] have written about how researchers fail to use the most appropriate analytic tools when communicating with their audiences. Panter and Sterba [43] write, “It is well known that accessible statistical guidance given without an ethical imperative…is painfully slow to infiltrate applied practice (pg 2).” We believe this is the case with the practice of dichotomizing and discretizing continuous independent variables. We have provided two examples that are relevant to the field of nutrition: predicting how accurately someone can read a nutrition food label and predicting fruit and vegetable intake. We recommend that researchers who publish in journals like *Nutrition and Diabetes* refrain from losing information about BMI levels when studying its relationship with health and nutrition knowledge variables through procedures like dichotomization and discretization.
